# MiRNAs: a new target for Chinese medicine to repair the intestinal barrier in the treatment of ulcerative colitis

**DOI:** 10.3389/fphar.2024.1446554

**Published:** 2024-08-09

**Authors:** Dajuan Sun, Zhongtao Zhang, Jingwei Xue

**Affiliations:** ^1^ The Affiliated Taian City Central Hospital of Qingdao University, Taian, China; ^2^ Shandong University of Traditional Chinese Medicine, Jinan, China

**Keywords:** ulcerative colitis, intestinal mucosal barrier, MicroRNAs, mechanism of action, traditional Chinese medicine

## Abstract

Ulcerative colitis (UC) is a chronic nonspecific inflammatory bowel disease whose pathogenesis remains unclear. Dysfunction of the intestinal mucosal barrier is closely related to the pathogenesis of UC, which is characterised by damage to the colon epithelial barrier, disruption of immune homeostasis, and persistent inflammatory cell infiltration. MicroRNAs (miRNAs) exhibit specific or differential expression in both UC animal models and patients, implicating their involvement in the pathogenesis of UC. In recent years there has been progress in using Traditional Chinese medicine (TCM) to regulate miRNA expression for repairing the intestinal mucosal barrier in UC, as demonstrated in animal and cell experiments. However, it has not been applied in a clinical setting and its underlying molecular mechanisms require further investigation. Therefore, this study systematically described the role of miRNAs in UC-induced intestinal barrier damage and the application of TCM to repair this intestinal barrier by regulating miRNA expression, offering new therapeutic targets for UC treatment.

## Graphical Abstract

**Figure:**
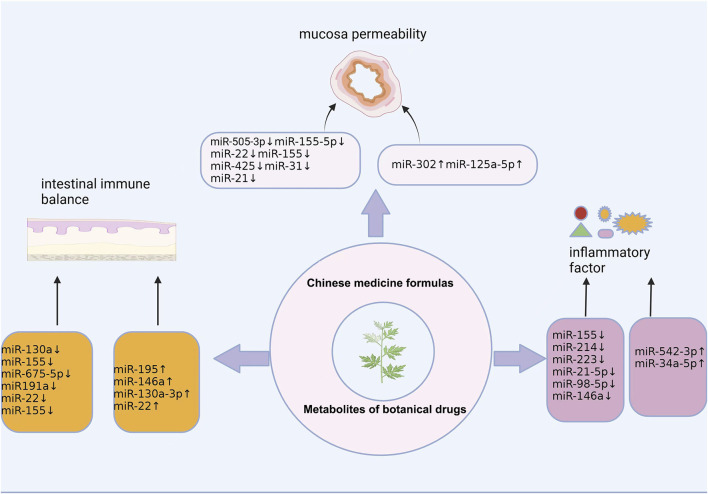
This review presents the progress of traditional Chinese medicines in the treatment of ulcerative colitis by regulating miRNAs.

## 1 Introduction

Ulcerative colitis (UC) is a chronic nonspecific inflammatory bowel disease involving the rectum and colon that invades the intestinal mucosa and submucosa. The disease is recurrent and difficult to treat. In recent years, the prevalence of UC has increased and poses a substantial disease burden in China (Nagao-Kitamoto et al., 2022; Le Berre et al., 2023). Modern medicine does not clarify the pathogenesis of UC. Further research reported that intestinal mucosal barrier function is impaired in the pathogenesis of UC, which is primarily characterized by colon epithelial barrier damage, destruction of immune homeostasis, and continuous inflammatory cell infiltration (Le Berre et al., 2023).

In recent years, several studies have confirmed that micro-ribonucleic acids (miRNAs) plays important roles in the occurrence and development of UC. miRNAs play a regulatory role in both the intestinal epithelial barrier and the intestinal mucosal immune system, with abnormal miRNA expression closely related to the pathogenesis of UC (Tian et al., 2019; Zhou et al., 2021). Furthermore, miRNAs have emerged as biological markers and potential therapeutic targets in UC research.

Numerous studies have shown that TCM has unique advantages in the treatment of UC, which can reduce the risk of UC recurrence and cancer, reduce hormone dependence, and improve the patient’s quality of life. Studies have demonstrated that botanical drugs and their metabolites can improve intestinal epithelium damage, regulate immunity, inhibit colon inflammation, and repair the intestinal mucosal barrier in UC by regulating the expression of miRNAs (Zhang X. et al., 2024; Wang et al., 2024). However, these therapies have not been applied in clinical practice. Therefore, this paper summarises the mechanism of miRNAs in UC and reviews the therapeutic effects and molecular mechanisms of TCM in modulating miRNA expression in UC. This aims to provide novel insights for clinical research on UC.

## 2 miRNA and UC intestinal mucosal barrier

### 2.1 Overview or miRNAs

miRNAs are endogenous single-stranded non-coding RNAs that are highly conserved across most organisms. They typically consist of 17–25 nucleotides (nt) and play crucial roles in RNA silencing and post-transcriptional regulation of gene expression. Studies have shown that miRNAs are involved in the regulation of cell proliferation, apoptosis, and differentiation, as well as in the pathogenesis of inflammatory diseases (Jung et al., 2021). miRNA synthesis is a multi-step process. First, the intron or gene spacer region (IGR) in the gene-coding region is transcribed by RNA polymerase II to generate the primary miRNA (pri-miRNA). The pri-miRNA is cleaved by the type III ribonuclease enzyme Drosha to form a precursor miRNA (pre-miRNA) approximately 70 nucleotides in length with a stem ring structure. The pre-miRNA is subsequently transported to the cytoplasm by the transporter Exportin-5 and processed by the type III ribonuclease Dicer to form mature double-stranded miRNAs. One miRNA binds to the argonaute protein with endonuclease activity to form an RNA-induced silencing complex (RISC) (Takahashi et al., 2018; Rawat et al., 2019). miRNAs in the RISC bind to the 3′UTR of mRNA mainly through the seed sequence (Kim et al., 2017), causing mRNA degradation or blocking the translation process, thereby reducing target gene expression. In addition, miRNA binding sites have also been found in other regions of mRNA, including the 5′UTR, pri-miRNA, coding sequence, and promoter regions (Tang et al., 2012; 2017; Kalantari et al., 2017). miRNAa are involved in almost all biological processes within eukaryotic cells and are stably present in almost all body fluids, often encapsulated in extracellular vesicles, thus exerting a remote regulatory role (Ma et al., 2018).

miRNAs are abnormally expressed in many diseases, including UC. In UC, they regulate its pathogenesis and influence the occurrence and development of colon tumours. For example, the expression of miR-18a and miR-19a is elevated in colitis-associated colon cancer (CAC) (He et al., 2016; Wang et al., 2017). According to literature reports (Takagi et al., 2010; Wang et al., 2019; Alrafas et al., 2020; Guo et al., 2022; Jimenez et al., 2022), miR-301a, miR-146a, miR-31, miR-132, miR-21, miR-155, miR-214, and other miRNAs were significantly upregulated in UC. The expression of miR-181 and miR-148a-3p were downregulated. Furthermore, miR-214 had a bidirectional regulatory effect (Li et al., 2017; Liu et al., 2019). Thus, miRNAs are involved in the pathogenesis of UC through multiple targets and pathways. These miRNA studies provide a new perspective on the molecular mechanisms of UC, and further investigation on the exact role of miRNAs in UC are required to explore new targets for UC therapy.

### 2.2 UC intestinal mucosal barrier

The intestinal mucosal barrier is composed of intestinal microorganisms, mucus, epithelial cells, secretory immunoglobulin A (sIgA), and intestinal-associated lymphoid tissue (GALT). It is the first line of defence to prevent external antigens, such as microorganisms, allergens, and carcinogens, from invading the intestinal mucosal tissue and is considered the upstream mechanism that leads to impaired immune balance (An et al., 2022). Abnormal structure and permeability of the adhesion and tight junctions of the intestinal mucosal epithelium lead to impaired intestinal mucosal barrier function. External antigens penetrate the body through the damaged intestinal mucosal barrier, triggering an immune response that activates many pro-inflammatory factors. This aggravates the intestinal mucosal inflammatory response, further compromising the barrier function. The intestinal epithelial barrier, immune response, and inflammatory infiltration complement each other and are interlinked, leading to the occurrence and development of this condition.

## 3 The role of miRNA in the injury of UC intestinal mucosal barrier (Figure 1)

### 3.1 miRNA disruption of the intestinal epithelial barrier is an early event in UC intestinal

The intestinal epithelial barrier comprises tightly connected intestinal epithelial cells (IEC). Different types of specialised epithelial cells, such as intestinal cells, panel cells, goblet cells, intestinal endocrine cells, and microfolded cells, constitute the intestinal upper cortex. These cells are renewed by pools of LGR5 intestinal stem cells (ISC) residing in the intestinal crypts (Gehart and Clevers, 2019). Tight junctions (TJ) consist of transmembrane proteins such as occludin, sealing protein (claudin), junction adhesion molecule (JAM), and scaffold proteins such as ZO-1, ZO-2, ZO-3, which connect transmembrane proteins to the cytoskeleton. In particular, tight junctions at the top of the cell play an important role in the regulation of mucosal permeability (Liang et al., 2023). Damage to the epithelial barrier and structural defects, including the decreased expression of tight junction proteins such as claudin and occluding and increased mucosal permeability, are important pathogenic factors of UC.

**FIGURE 1:**
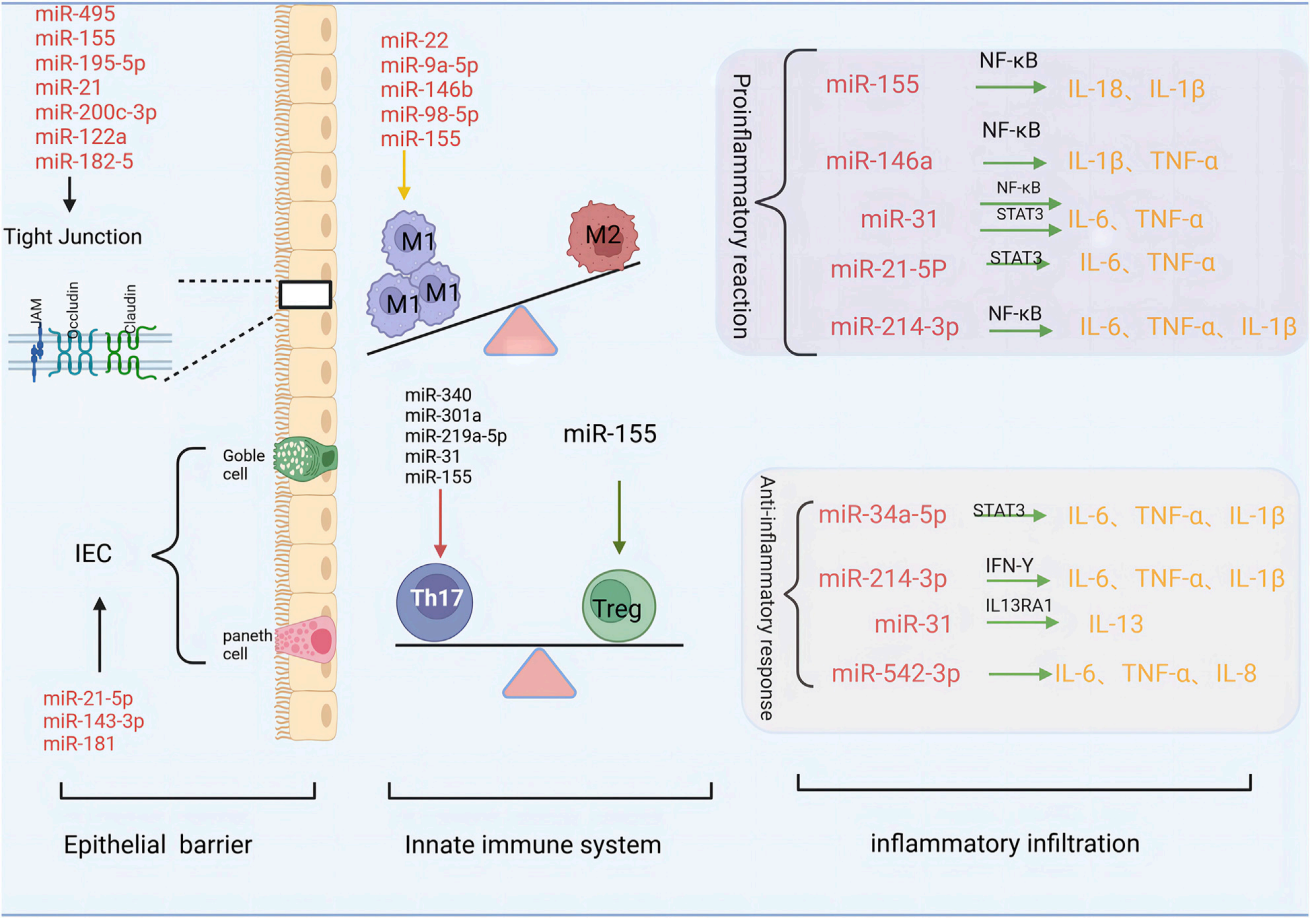
The role of miRNA in the injury of UC intestinal mucosal barrier

An increasing number of studies have identified miRNAs as key regulators of the intestinal epithelial cell barrier, which in turn regulates IEC growth and apoptosis (Zhou et al., 2021). Human umbilical cord mesenchymal stem cell exosomes (HucMSC-exo) downregulate key miRNAs such as miR-21-5p and miR-143-3p, activate the Wnt/β-catenin signalling pathway, upregulate the expression of LGR5, and promote the regeneration of ISC and intestinal epithelium, thereby alleviating experimental colitis (Liang et al., 2023). Jimenez et al. (Jimenez et al., 2022) reported that miR-181 downregulation promoted the development of severe colonic inflammation in IEC of patients with inflammatory bowel disease (IBD) and dextran sulphate (DSS) -induced colitis in mice, suggesting that miR-181 may affect epithelial barrier function by regulating IEC proliferation and cell metabolism.

In addition, miRNAs regulate tight junctions to maintain intestinal epithelial barrier function. Chu et al. (Chu et al., 2018) reported that miR-495 may improve intestinal mucosal barrier function in UC mice by inhibiting the JAK/STAT3 signalling pathway and up-regulating Claudin-1 expression. In a DSS-induced colitis model, miR-155 antagonists reduced intestinal barrier damage and TJ protein loss by inducing the expression of hypoxia-inducible factor 1 (HIF-1) (Liu et al., 2020). Furthermore, miR-195-5p protected intestinal permeability by regulating the expression of Claudin-2 and other TJ proteins, thereby repairing the intestinal epithelial barrier in UC mice (Scalavino et al., 2022). In contrast, miR-301a reduces the expression of cadherin-1 by targeting BTG anti-proliferation factor 1 (BTG1) and increases cell permeability to disrupt intestinal barrier function and promote inflammation (He C. et al., 2017). MiR-200c-3p binds to the 3′UTR non-coding region of the occludin mRNA, inducing its breakdown and depleting occludin, thereby facilitating increased intestinal permeability (Rawat et al., 2020). Additionally, miR-182-5p, miR-21, and miR-122a upregulate intestinal inflammation, inhibit tight junction protein expression in UC colon tissue, enhance intestinal mucosal permeability, and disrupt the stability of the intestinal epithelial barrier (Zhang et al., 2015; 2017; Xu Y. et al., 2022), suggesting that they are potential targets for UC treatment.

### 3.2 miRNAs modulate immune cell dysregulation as a key component in the formation of UC

In recent years, an increasing body of evidence has demonstrated the involvement of miRNAs in immune cell differentiation, regulation, and signal pathways. Moreover, they have been shown to exert regulatory effects on macrophage polarization and T cell proliferation while also playing a crucial role in maintaining intestinal homeostasis among patients with UC. Damage to the colonic mucosal barrier caused by immune system disorders is a key link in the occurrence and development of UC (Xu M. et al., 2022). During the development of UC, the innate immune system senses the inflammatory environment generated by foreign microbiota through NOD-like receptors (NLR) and pattern recognition receptors (PRR). NLRs and PRRs are present in innate immune cells such as macrophages, neutrophils, and dendritic cells (DCS) (Ahluwalia et al., 2018). Persistent activation of the innate immune response drives pathogenic T cell responses (Na et al., 2019).

Macrophages are key effector cells involved in innate and adaptive immunity and can be divided into classically activated macrophages (M1) with a pro-inflammatory phenotype and alternately activated macrophages (M2) with an anti-inflammatory phenotype. Important metabolic changes occur in the intestinal macrophages of patients with UC; excessive activation and infiltration of macrophages leads to intestinal damage, suggesting that macrophages are the dominant cell type involved in the pathogenesis of UC. In the UC developmental stage, macrophages predominantly exhibit the M1 phenotype, secreting pro-inflammatory factors that participate in the development of UC alongside the pro-inflammatory mediators they produce. Recent studies have revealed that miRNAs are key players in the regulation of M1-type polarisation in macrophages (Essandoh et al., 2016). miR-98-5p promotes the M1 polarisation of macrophages, with experiments demonstrating that miR-98-5p knockout increases the expression of Trib1, which polarises M1 macrophages to the M2 phenotype (Peng et al., 2020). Similarly, miR-146b inhibits M1 macrophage activation by inhibiting the Toll-like receptor 4 (TLR4) signalling pathway, thereby inhibiting the release of pro-inflammatory cytokines (Deng et al., 2019). In addition, microRNA-155, miRNA-9a-5p, and miR-22 are also involved in cell metabolism, macrophage proinflammatory response, and M1 polarization (Pasca et al., 2020; Kang, 2023; Li et al., 2023), suggesting that regulation of related miRNA-mediated macrophage inflammatory response may be an option for the treatment of IBD.

Evidence suggests that the balance between Th17 and regulatory T cells (Tregs) is critical for preventing pathogen invasion and inhibiting excessive effector T-cell responses in the intestinal mucosa (Zhang et al., 2018). Xu et al. (2017) showed that miR-155 is significantly upregulated in the colon tissue of patients with active UC. Subsequent animal experiments confirmed that miR-155 induces Th17 differentiation by targeting JARID2, thus playing a role in host damage. Moreover, Zhu et al. (2020) discovered that silencing miR-155 not only inhibits Th17 differentiation by targeting JARID2, but also increases sfrp1 and activates the Wnt signalling pathway to regulate Tregs, thereby maintaining Th17/Treg cell balance and alleviating DSS-induced colitis. In a mouse model of colitis, Chen et al. (2020) reported that miR-340 promotes Th17 differentiation by targeting scaffold/stroma-associated region-binding protein 1 (SMAR1). *In vitro* studies using samples from mouse colitis models demonstrated that miR-301a, miR-219a-5p, and miR-31 inhibits Th1/Th17-mediated immune response and intestinal inflammation (He et al., 2016; Shi et al., 2017; 2020).

### 3.3 miRNAs regulate cytokine superfamily release as an important mediator of UC intestinal

Cytokines play an important role in exacerbating and continuing UC through signal transduction. They drive inflammation and the pathogenesis of UC by producing inflammatory mediators and activating inflammatory pathways (Tatiya-Aphiradee et al., 2018). The colon tissue of patients with active UC is infiltrated by activated immune cells, which triggers a cascade reaction that eventually leads to overexpression of pro-inflammatory cytokines such as TNF-α, IL-6, IL-1β, IFN-Y, IL-17A, and IL-18 and the downregulation of anti-inflammatory cytokines such as TGF-β, IL-10, IL-13 (Friedrich et al., 2019). However, miRNAs can regulate the release of these inflammatory factors, causing an imbalance in pro-inflammatory and anti-inflammatory cytokines. This leads to intestinal mucosal barrier damage, thereby contributing to the development of UC.

Thus, miR-146a, miR-155, and miR-21-5p may play pro-inflammatory roles. Studies have shown that miR-146a activates the NF-κB pathway by targeting TAB1 to promote apoptosis and colonic inflammation (Xia et al., 2024), whereas silencing miR-155 leads to the downregulation of inflammatory cytokines, such as IL-1β and IL-18, by reducing the expression of pNF-κB protein (Zeng et al., 2020). Furthermore, the overexpression of miR-155 leads to increased NF-κB activity and serum IL-6 levels for an extended duration (Mann et al., 2018). Lu et al. (2020) discovered that the expression of miR-21-5p was significantly increased in the serum of patients with UC and in the colon tissue of UC rats. Furthermore, the downregulation of miR-21-5p mediates the IL-6/STAT3 pathway to reduce the levels of IL-6 and TNF-α, thereby reducing the levels of inflammation and apoptosis of RAW264.7 cells.

miR-34a-5p and miR-542-3p play anti-inflammatory roles. Chen et al. (2024) found through transfection experiments that the overexpression of miR-34a-5p can reduce the levels of IL-6, IL-1β, and TNF-α, and miR-34a-5p may reduce the inhibition of the release of pro-inflammatory factors by regulating theIL-6/STAT3 pathway, thereby improving the level of inflammation and relieving UC. The experimental results of Gong et al. (2022) showed that the activity of IL-8, IL-6 and TNF-α could be effectively inhibited by upregulation of the expression of miR-542-3p, and the inflammatory response could be reduced.

miR-31 and miR-214 have bidirectional regulatory effects owing to their different target genes and downstream pathways. miR-31 is upregulated in patients with UC and mice with colitis STAT3 and NF-κB activate the transcription of miR-31 in colorectal cancer cells and organoids, and miR-31 prevents the expression of inflammatory cytokine receptors (IL17R and IL17RA) and signalling protein (GP130). Thereby reducing the levels of TNF-α and IL-6 and exerting anti-inflammatory effects (Tian et al., 2019). On the other hand, miR-31 directly targets the 3′UT of IL-13’s main receptor (IL13RA1) mRNA, thereby blocking IL-13 signalling and affecting the host’s anti-inflammatory ability (Gwiggner et al., 2018). Chen et al. (Chen X. et al., 2021) interfered with DSS induced mice and found that while the expression of miR-214 decreased, the levels of serum inflammatory factors IL-1β, IL-6 and TNF-α decreased. miR-214-3p can not only inhibit the expression of IFN-γ and intestinal inflammation (Li et al., 2017), but also activate the NF-κB pathway and promote intestinal inflammation (Liu et al., 2019).

Thus, miRNAs play important roles in the inflammatory process in UC. Multiple miRNAs participate in and affect the release of inflammatory cytokines and are important mediators in regulating the release of the cytokine superfamily, causing damage to the intestinal mucosal barrier in UC.

## 4 TCM regulates the application of miRNA in the injury of UC intestinal mucosal barrier

According to the clinical symptoms of UC, it can be classified as the “dysentery” of Chinese medicine. TCM treatment of UC has good clinical efficacy and fewer adverse reactions, and can also improve the quality of life of patients and prevent complications. Hence, in recent years, TCM treatment of UC has become a research hotspot. Many experiments have verified the intervention effect of TCM on UC and its mechanism of action. The mechanism of TCM in the treatment of UC by regulating miRNAs has also been verified. Botanical drugs and their metabolites repair intestinal epithelial barrier damage, regulate the intestinal immune balance, improve intestinal inflammation in UC, and repair the intestinal mucosal barrier through miRNA regulation.

### 4.1 Reduce colon permeability and repair the integrity of intestinal mucosal barrier

#### 4.1.1 Chinese medicine formulas

Shenlingbaizhu San (SLBZS) comes from the ‘Prescriptions Collected by the Public Pharmacy’. It is composed of 12 botanical drugs such as *Panax ginseng* C.A.Mey. [Araliaceae; Ginseng radix et rhizoma], *Poria cocos* (Schw.) Wolf [Polyporaceae; Poria], *Atractylodes macrocephala Koidz.* [Asteraceae; Atractylodis macrocephalae rhizoma], etc. Zhu (Zhu Y., 2022) used SLBZS to intervene in DSS-induced mice, and mesalazine was used as a positive control. The results suggested that SLBZS significantly inhibited weight loss; improved disease activity (DAI), colon shortening, and pathological injury; and downregulated the expression of miR-130a. The increased expression of peroxisome proliferator-activated receptors (PPARγ) and occludin improves intestinal permeability. This trend was most evident in the medium-dose group. Wei et al. (2017) used dual luciferase activity to determine that miR-130a directly targeted PPARγ, and miR-130a inhibitors enhanced the expression of PPARγ. After activation of PPARγ, SLBZS can protect intestinal barrier by increasing the production of Occludin (Wang et al., 2012; Wang L. and Wang J., 2022), which indicates that SLBZS may improve intestinal mucosal barrier through the miR-130a/PPARγ/occludin pathway and then improve UC.

Kaixuan Decoction (KXT) is a Chinese medicine formulas created by Professor Yue Rensong. It is composed of 18 botanical drugs, including *Codonopsis pilosula* (Franch.) Nannf. [Campanulaceae; codonopsis radix], *Hansenia weberbaueriana* (Fedde ex H. Wolff) Pimenov & Kljuykov [Apiaceae; notopterygii rhizoma et radix], *Angelica biserrata* (R.H.Shan & C.Q.Yuan) C.Q.Yuan & R.H.Shan [Apiaceae; angelicae pubescentis radix], etc. Qiu et al. (2022) treated patients with mild UC with subtilis enteric-coated capsules combined with salazopyridine enteric-coated tablets as the control group, based on which KXT was added. In the observation group, the research results showed that KXT significantly reduced the TCM syndrome score of patients and improved the Mayo activity index. KXT reduced the levels of D-LA, BT, and DAO in the peripheral blood, downregulated the expression of miR-155, and upregulated the expression of miR-195. Studies have shown that increased levels of D-LA, BT, and DAO in peripheral blood can affect intestinal mucosal permeability and damage the intestinal mechanical barrier (He Z. et al., 2017). This suggests that KXT may alter mucosal permeability, repair the intestinal mucosal barrier, and treat UC by downregulating and upregulating miR-155. However, its specific mechanism of action remains to be further explored.

Fufangkushen Decoction (FFKSD) is composed of 6 botanical drugs, including *Sophora flavescens* Aiton [Fabaceae; sophorae flavescentis radix], *Sanguisorba officinalis* L. [Rosaceae; carbonized sanguisorbae radix], *Bletilla striata* (Thunb.) Rchb. f. [Orchidaceae; bletillae rhizoma], etc. Wu (2022) used FFKSD to interfere with DSS-induced mice (3.64 g/kg, 7.28 g/kg, 14.56 g/kg for 7 days), using mesalazine as the positive control drug. The results showed that FFKSD significantly improved the body weight and colon length of the model mice and reduced the DAI and histological scores. Moreover, it inhibited the expression of Notch1 and hairy enhancer of split (Hes 1) and upregulated the expression of miR-146a, ATOH1, and MUC2. Notch1 was a direct target of miR-146a. Stimulated and activated Notch pathways can upregulate the expression of the transcription factor Hes1, and the transcription of ATOH1, as the downstream target gene of Hes1, is inhibited, which leads to the inhibition of the differentiation of secretory cell lines such as goblet cells, a decrease in mucin expression such as MUC2, and thinning of the mucus layer (Artavanis-Tsakonas et al., 1999). This study indicates that FFKSD may inhibit the Notch signalling pathway by upregulating the expression of miR-146a, upregulating the expression of downstream ATOH1, inhibiting overactivation of the Notch signalling pathway, promoting goblet cell differentiation and mucous secretion, and repairing the intestinal mucosal barrier function in UC.

Qingchangwenzhong Formula (QCWZF) is composed of *Coptis chinensis* Franch. [Ranunculaceae; coptidis rhizoma], *Zingiber officinale* Roscoe [Zingiberaceae; zingiberis rhizoma praeparatum], *Strobilanthes cusia* (Nees) Kuntze [Acantharean; indigo naturalis], *S. flavescens* Aiton [Fabaceae; sophorae flavescentis radix], etc. Sun et al. (2019) found that QCWZF interfered with DSS-induced mice, and found that QCWZF downregulated the expression of miR-675-5p, and increased the expression of VDR mRNA in the colon tissue. Enhancing the expression of ZO-1 and occludin in the colon tissue significantly reduced the level of serum IL-17 and increased the level of serum IL-10. VDR, a member of the steroid hormone/thyroid hormone receptor superfamily, can specifically bind to vitamin D, regulate the levels of vitamins D1 and 25(OH)2D3, alter epithelial permeability, and promote the expression of TJ proteins. This suggests that QCWZF enhances intestinal mucosal barrier function via the miR-675-5p/VDR signalling pathway.

#### 4.1.2 Metabolites of botanical drugs

Baicalin, a flavonoid extracted from *Scutellaria baicalensis* Georgi [Lamiaceae; scutellariae radix], has anti-inflammatory, antioxidant, antiviral, and other pharmacological effects (Zhang J. et al., 2024). Wang (2018) supplied Baicalin to TNF-α-induced IEC-6 cells. The results showed that baicalin improved cell survival rate, decreased cell apoptosis, increased transmembrane resistance, increased ZO-1 protein expression, decrease Claudin-2 protein expression, and downregulated miR-191a expression. The transfection of miR-191a mimics and inhibitors into TNF-α-induced IEC-6 cells confirmed that ZO-1 was the target gene of miR-191a. Furthermore, miR-191 inhibitors enhanced the protective effect of Baicalin on ZO-1 and accelerated cell migration. This study showed that baicalin downregulated miR-191a. By targeting ZO-1, Baicalin reduces colon permeability and restores the integrity of the intestinal epithelial barrier.

Icariin (ICA) is a natural flavonoid extracted from the *Epimedium sagittatum* (Siebold & Zucc.) Maxim. [Berberidaceae; epimedii folium], which has pharmacological effects such as enhancing immunity, anti-tumour, and anti-osteoporosis (Qi and Zheng, 2022). Li (2023) supplied ICA to TNF-α-induced Caco-2 monolayer cells and demonstrated that ICA increased the TEER value, decreased 4-KD FITc-glucan (para-cellular permeability tracer) passing rate, restored linear fluorescence signal of occludin, downregulated the expression of miR-122, and upregulated the expression of occludin mRNA. After transfection with miR-122a mimics, the ability of ICA to repair the intestinal mucosal barrier was weakened. This suggests that ICA reduces colonic permeability and repairs the intestinal mucosal barrier by down-regulating the expression of miR-122a.

Matrine, a natural alkaloid contained in *S. flavescens* Aiton [Fabaceae; sophorae flavescentis radix], has anti-inflammatory, anti-tumour, anti-oxidation, and anti-viral effects (You et al., 2020). Yu et al. (2023) showed that matrine can increase the TEER value, reduce dexosan permeability, upregulate the expression of occludin and ZO-1 proteins, and downregulate the expression of ROCK1 and miR-155 in both an inflammatory model of Caco-2 cells overexpressing miR-155 and in DSS-induced mice. Overexpression of miR-155 reverses the effects of matrine. This study suggests that matrine may protect the intestinal barrier from dysfunction by downregulating the expression of miR-155, inhibiting the Rho/Rock pathway-associated protein ROCK1, and maintaining the TJ.

Berberine (BBR) is an metabolite rich in *C. chinensis* Franch. [Ranunculaceae; coptidis rhizoma], *Phellodendron chinense* C.K.Schneid. [Rutaceae; phellodendri chinensis cortex], ect. Its anti-inflammatory, anti-apoptotic, anti-tumour, blood pressure-lowering, and blood sugar-lowering effects have been widely studied (Ai et al., 2022). Zhao (2023) administered BBR through gavage to DSS-induced mice demonstrating that BBR reduced glucan permeability, increased occludin mRNA expression in UC mice, upregulated the expression of miR-103a-3p, and downregulated the expression of Bromodomain-containing protein 4 (BRD4). BRD4 belongs to the bromine family of proteins. Chen L. et al. (2021) have found that inhibition of BRD4 can block LPS-induced colonic TJ barrier dysfunction, cell pyroptosis, and inflammation. Subsequent experiments verified the relationship between miR-103a-3 and BRD4 expression. In addition, BBR, elevated miR-103a-3p, or inhibited BRD4 could significantly inhibit the Wnt/β-catenin pathway. In this study, BBR plays a therapeutic role by up-regulating the expression of miR-103a-3p. It targets BRD4 and inhibits the activation of Wnt/β-catenin pathway, upregulates the expression of occludin, and repairs the intestinal mucosal barrier.

Naringenin (NAR) is the main flavonoid component of *Citrus reticulata* Blanco [Rutaceae; citri reticulatae pericarpium], *Citrus × aurantium* f. aurantium [Rutaceae; aurantii fructus immaturus], and other botanical drugs. It has anti-inflammatory and antioxidant activities (Tseng et al., 2021). Xie et al. (2021) studied DSS-induced rats and found that NAR improved the DAI, gross mucosal score, and histopathological score of DSS-induced rats. Transmission electron microscopy revealed that the intestinal mucosal barrier was damaged, desmosomes disappeared, and cell gap widening was significantly alleviated. NAR can upregulate the expression of miR-22, increase the expression of ZO-1, occludin, and claudin-1 proteins, and inhibit the expression of NLRP3 protein, and the application of miR-22 antagomir can reverse these changes. Furthermore, double luciferase reporter gene experiments verified the relationship between miR-22 and NLRP3, suggesting that NAR could upregulate the expression of miR-22, inactivate the NLRP3 inflammasome, increase the expression of TJ proteins, repair the intestinal epithelial barrier, and thus treat UC.

These studies have demonstrated that botanical drug metabolites can enhance intercellular TJs, and enhance intestinal mucus secretion, by regulating specific miRNAs, so as to repair the permeability and integrity of the intestinal mucosal barrier, and protect the first line of defence against pathogen invasion, which is in line with the concept of TCM that “when positive qi exists in the intestinal system, the evils cannot be interfered with”.

### 4.2 Regulate intestinal immune balance and reduce intestinal mucosal barrier damage

#### 4.2.1 Chinese Medicine Formula

Sishen Pill (SSP) composed of 6 botanical drugs, including *Cullen corylifolium* (L.) Medik. [Fabaceae; psoraleae fructus], T*etradium ruticarpum* (A.Juss.) T.G.Hartley [Rutaceae; euodiae fructus], *Myristica fragrans* Houtt. [Myristicaceae; myristicae semen], *Schisandra chinensis* (Turcz.) Baill. [Schisandraceae; schisandrae chinensis fructus], *Z. officinale* Roscoe [Zingiberaceae; zingiberis rhizoma recens], *Ziziphus jujuba* Mill. [Rhamnaceae; jujubae fructus]. Huang (2024) used SSP to interfere with DSS-induced mice, and the results showed that SSP significantly reduced body weight, FOB score, DAI score, and histopathological score and restored the spleen weight index, colon length, colon weight, and intestinal weight index of DSS-induced mice. In addition, flow cytometry showed that SSP could effectively inhibit the activation, maturation, and proliferation of dendritic cells (DC). In mouse bone marrow-derived dendritic cells (BMDCs) cultured *in vitro*, SSP simultaneously inhibited miR-505-3p and E-cadherin expression in a dose-dependent manner. However, after inhibition with transfection of Lv-miR-505-3p-inhibition, the expression of miR-505-3p and E-cadherin decreased uniformly, confirming the relationship between the two. E-cadherin and DCs accumulate in large quantities at the site of UC, highly express toll-like receptors, release inflammatory cytokines such as IL-6 and IL-23, and increase the response of Th17 cells in the intestine, thus aggravating colitis (Siddiqui et al., 2010). This indicates that SSP can inhibit the expression of E-cadherin by downregulating miR-505-3p, avoiding the amplification of inflammatory cascades, regulating intestinal immune homeostasis, and achieve the purpose of treating ulcerative colitis.

Xiezhuojiedu Formula (XZJDF) is an empirical Chinese Medicine Formula, composed of 10 botanical drugs, including *Houttuynia cordata* Thunb. [Saururaceae; houttuyniae herba], *Plantago ovata* Forssk. [Plantaginaceae; plantaginis semen], *S. baicalensis* Georgi [Lamiaceae; scutellariae radix], ect. Sun et al. (2024) found that the upregulation of miR-155 may be key to the occurrence of UC. A key DEGs in UC is SOCS1, whose main downstream transcription factor is STAT3. Subsequently, XZJDF was administered to DSS-induced mice, and mesalazine was used as a positive control. The results showed that XZJDF could improve symptoms such as diarrhoea and hematochezia, effectively inhibit weight loss, and reduce DAI and histopathological scores. This effect was better than that of mesalazine. Additionally, XZJDF significantly downregulated the expressions of miR-155, p-STAT3, p-JAK2, and ROR-γt proteins in colon tissue of mice, upregulated the expression of SOCS1 protein, significantly decreased the levels of serum IL-17 and IL-6, and increased the levels of IL-10 and TGF-β. Studies have shown that in the presence of p-STAT3, the level of Th17 cell-specific transcription factor ROR-γt is upregulated, which promotes the shift of CD4+T cells towards Th17 cell differentiation (Chen and Fan, 2016; Crawford et al., 2020), suggesting that XZJDF can downregulate the expression level of miR-155-5p. This consequently targets the JAK2/STAT3/SOCS1 signalling pathway, inhibits the differentiation of CD4+T cells to Th17 cells, and plays a role in regulating immunity and maintaining a balance in inflammation.

Qingchang Huayu Formula (QCHYF) is composed of 17 botanical drugs, including *Pulsatilla chinensis* (Bunge) Regel [Ranunculaceae; pulsatillae radix], *C. chinensis* Franch. [Ranunculaceae; coptidis rhizoma], *Fraxinus excelsior* L. [Oleaceae, fraxini cortex], ect. Yang et al. (2022) used mesalazine to treat patients with large intestine damp heat-type UC as a control group and mesalazine tablets and Qingchang Huayu decoction as the research group. The results showed that DAI, and Geboe index scores of the study group were all lower than those of the control group, and the quality of life of the patients was significantly improved. QCHYF reduces the number of Th17 cells in peripheral blood and downregulates the expression of miR-22 Studies have shown that Th17 cells belong to the CD4+T cell subgroup, and the inflammatory factors secreted by TH17 and IL-21 can destroy the barrier function of the intestinal mucosa, induce the aggregation of inflammatory cells, and lead to or aggravate the local immune inflammatory response. Moreover, it promotes the occurrence and progression of UC (Yan et al., 2020). This suggests that QCHYF may treat UC by downregulating miR-22 and inhibiting the inflammatory differentiation of Th17 cells. However, the specific mechanism of action remains to be further explored.

Jiuwei Baizhu Decoction (JWBZD) is composed of 9 botanical drugs: *Pueraria montana* var. lobata (Willd.) Maesen & S.M.Almeida ex Sanjappa & Predeep [Fabaceae; puerariae lobatae radix], *Sargentodoxa cuneata* (Oliv.) Rehder & E.H.Wilson [Lardizabalaceae; sargentodoxae caulis], *Codonopsis pilosula* (Franch.) Nannf. [Campanulaceae; codonopsis radix], ect. Yang et al. (2023) administered JWBZD to TNBS/ethanol-induced rats and found that JWBZD downregulated the expression of miR-155, promoted the expression of SOCS-1 protein, inhibited the expression of STAT3 protein, and decreased the levels of TNF-α, IL-23, IL-6, IL-1β, and other inflammatory factors. However, miR-155 overexpression reversed the efficacy of JWBZD. The findings revealed that increased miR-155 expression in UC aggravated the disease process. miR-155 affects the function of multiple immune cells through its target genes. As a downstream negative regulator, SOCS-1 negatively regulates the STAT pathway and inhibits signal transduction of IL-6 and other cytokines (Kimura et al., 2005). This study showed that JWBZD promoted the expression of the SOCS-1 protein and regulated the STAT pathway by inhibiting the expression of miR-155, thereby inhibiting the signal transduction of pro-inflammatory factors and the expression of intestinal mucosal immune function factors, thus achieving a protective effect against diseases.

The composition of FFKSD has been mentioned above, Zhu F. (2022) used FFKSD and an miR-155 antagomir to treat DSS-induced mice; mesalazine was used as a positive control. The results showed that all three UC mice groups exhibited an improved DAI score, colon length, and colonic visual damage score and a decreased expression of miR-155. Among them, the miR-155 antagomir had the best effect, and it was observed under a fluorescence microscope that the miR-155 antagomir reached and penetrated the intestinal mucosal tissue. In addition, flow cytometry of spleen and mesenteric lymph nodes of UC mice showed that FFKSD and miR-155 antagomir decreased the proportion of Th17 cells, increased the proportion of Tregs cells, and decreased the mRNA and protein expression levels of IL-6, IL-17A and RORγt. And increased the expression levels of TGF-β and IL-10. Subsequent experiments verified that FFKSD and miR-155 antagomir inhibited sfrp1 and promoted JARID2, Wnt1, β-catenin, TCF-4 and Cyclin D1. Studies have found that JARID2 can promote the transmission of Wnt/β-catenin signal by inhibiting SFRP1, thus regulating skeletal muscle differentiation (Adhikari and Davie, 2018). This suggests that FFKSD may regulate the expression of Th17 and Treg cells and their related inflammatory factors by down-regulating the expression of miR-155 and targeting the Jarid2/Wnt/β-catenin axis, thereby maintaining intestinal Th17/Treg balance and alleviating intestinal inflammation.

#### 4.2.2 Metabolites of botanical drugs

Curcumin is a polyphenol extracted from the botanical drug, *Curcuma longa* L. [Zingiberaceae; curcumae longae rhizoma], which has anti-inflammatory, antitumour, and other pharmacological activities (Laurindo et al., 2023). Song et al. (2020) used curcumin to treat TNBS-induced rats, and found that curcumin could effectively reduce DAI score, reduce the expression of miR-425 in peripheral blood, reduce the levels of pro-inflammatory factors IL-6 and IL-17, and increase the levels of anti-inflammatory factor transforming growth factor-β. Flow cytometry revealed a decrease in the percentage of Th17 cells and an increase in the percentage of Tregs. The Th17 cells are a subgroup of CD^4+^ T cells that secrete IL-17. IL-6 and TGF-β can jointly initiate the differentiation of CD^4+^ T into Th17 cells, and TGF-β can induce the differentiation of CD^4+^ T cells into Treg cells with mucosal protection by promoting the expression of Foxp3, thus triggering a cascade of inflammatory response. This study suggests that curcumin regulates the Th17/Treg balance and maintains intestinal immune homeostasis by down-regulating the expression of miR-425.

Resveratrol is a natural Phenols found in a variety of botanical drugs such as *Reynoutria japonica* Houtt. [Polygonaceae; polygoni cuspidati rhizoma et radix], *Smilax glabra* Roxb. [Smilacaceae; Smilacis glabrae rhizoma]. It has anti-inflammatory, antiviral, antioxidant, anticancer, and immune-regulatory activities (Harikumar et al., 2010). Alrafas et al. (2020) used resveratrol gavage to treat TNBS mice and found that resveratrol effectively improved clinical manifestations in UC mice. Meanwhile, flow cytometry showed that Th17 cells (CD4+IL-17+) and Th1 cells (CD4+IFNγ+) were reduced. The number of Tregs (CD4+FoxP3+) and IL-10-producing cells (CD4+IL-10+) increased. Subsequent microarray and principal component analyses (PCA) of CD4^+^ T cells showed that miR-31 plays a significant role in this process. Furthermore, transfecting miR-31 confirmed that miR-31 directly regulates the expression of Foxp3, suggesting that resveratrol targets the Foxp3 signal axis by downregulating the expression of mir-31, regulates the Th17/Treg balance, regulates immunity, maintains inflammation balance, and plays a role in the treatment of UC.

Alpinetin is a flavonoid extracted from *C. longa* L. [Zingiberaceae; curcumae longae rhizoma], *Wurfbainia compacta* (Sol. ex Maton) Škorničk. & A.D.Poulsen [Zingiberaceae; amomi fructus rotundus], *C. longa* L. [Zingiberaceae; curcumae radix] and other ginger plants. Alpinetin can repair cell damage and has anti-inflammatory and antibacterial properties. Lv et al. (2018) treated DSS-induced mice with different concentrations of alpinetin and used 5-ASA as a positive control. Alpinetin reduced the DAI score in a dose-dependent manner, improved body weight, colon length, and histopathological injury, and decreased MPO activity, a well-known marker of neutrophil infiltration. The efficacy of the alpinetin high-dose group was comparable to that of the 5-ASA group. In addition, alpinetin significantly increased the proportion of Treg cells and slightly decreased the proportion of Th17 cells in the mesenteric lymph nodes (MLNs) and lamina propria of the colon (LPs). *In vitro*, it improved Treg cell induction in a concentration-dependent manner but had no effect on Th17 cells. Subsequent experiments showed that alpinetin activated the aromatic hydrocarbon receptor (AhR), promoted the expression of miR-302, downregulated the expression of DNMT-1, reduced the methylation level of the Foxp3 promoter, promoted the binding of CREB to the Foxp3 promoter, upregulated Foxp3 expression, and regulated the Th17/Treg balance in the colon. This suggests that alpinetin ameliorates immune disorders, alleviates inflammatory infiltration, and improves colitis in mice by regulating miR-302, DNMT-1, and CREB signalling.

Cinnamaldehyde is the main metabolite extracted from *Cinnamomum verum* J. Presl [Lauraceae; cinnamomi cortex]. Previous studies have shown that it possesses antibacterial and anti-inflammatory properties (Lv et al., 2018). Qu et al. (2019) found that cinnamaldehyde improved clinical symptoms, shortened colon length and the degree of inflammatory cell infiltration, decreased the expression of pro-inflammatory factors, miR-21 and miR-155, in UC colon tissue, and downregulated the levels of phosphorylated AKT, mTOR, and COX2 proteins. miR-21 or miR-155 is associated with IL-1β and IL-6 in macrophage activation. These results suggest that cinnamaldehyde regulates the AKT/mTOR and COX2 pathways by inhibiting the expression of miR-21 and miR-155 in macrophages and plays a role in regulating immunity, alleviating inflammatory infiltration, and improving colitis in mice.

Diosgenin is a natural metabolite of botanical drugs extracted from *Dioscorea nipponica* Makino [Dioscoreaceae; dioscoreae nipponicae rhizoma], *D. nipponica* Makino [Dioscoreaceae; dioscoreae rhizoma], which has pharmacological effects such as anti-inflammatory, antioxidant, and immunity enhancement (Wang D. and Wang X., 2022). Shi et al. (2022) found that different concentrations of diosgenin decreased the polarisation of M1 macrophages in mice with colitis and upregulated the expression level of miR-125a-5p. *In vitro* experiments further showed that diosgenin inhibited the expression of the M1 marker gene (CD16) and enhanced the expression of M2 marker genes (CD206 and arginase-1), which were reversed by miR-125a-5p inhibitors. These results indicate that diosgenin regulates macrophage polarisation by upregulating miR-125a-5p, thereby improving UC.

In summary, some TCM monomers target the central components of the innate or adaptive immune system to avoid an immune overresponse by downregulating miR-31, miR-21, and miR-155 and upregulating miR-302 and miR-125a-5p, among other miRNAs that are abnormally expressed in patient tissues of the intestinal mucosal barrier exhibiting sustained damage.

### 4.3 Reduces inflammation levels and protects the mucosal membrane of the colon

#### 4.3.1 Chinese Medicine Formula

Gegen Qinlian Decoction (GQD) is composed of *P. montana* var. lobata (Willd.) Maesen & S.M.Almeida ex Sanjappa & Predeep [Fabaceae; puerariae lobatae radix], *C. chinensis* Franch. [Ranunculaceae; coptidis rhizoma], *S. baicalensis* Georgi [Lamiaceae; scutellariae radix], *Glycyrrhiza uralensis* Fisch. ex DC. [Fabaceae; Glycyrrhizae radix et rhizoma]. Gong et al. (2022) administered GQD to dinitrochlorobenzene (DNCB)/fethanol/acetic acid-treated rats, using mesalazine as a positive control. The results showed that GQD or mesalazine could effectively inhibit the activities of serum inflammatory factors IL-8, IL-6, and TNF-α and reduce the content of malondialdehyde in UC rats. It increased oxidative stress-related indices such as superoxide dismutase (SOD) and glutathione peroxidase (GSH-PX) in colon tissue, outperforming mesalazine at high doses, indicating that GQD has significant anti-inflammatory and antioxidant stress effects. In addition, GQD significantly upregulated the expression of miR-542-3p. Subsequent experiments verified that the inhibition of miR-542-3p reversed the effects of GQD on inflammatory mediators and oxidative stress in UC rat colon cells, suggesting that GQD inhibits inflammatory mediators by upregulating the expression of miR-542-3p. Furthermore, it reduced oxidative stress in UC rats.

Kuijieling Decoction (KJL) is comprised of 5 botanical drugs: Ilex rotunda Thunb. [Aquifoliaceae, ilicis rotundae cortex], *Atractylodes lancea* (Thunb.) DC. [Asteraceae, rhizoma atractylodis macrocephalae], *Paeonia lactiflora Pall.* [Paeoniaceae; Paeoniae radix rubra], Hirudo nipponica Whitman [Hirudinidae; Hirudo], *G. uralensis* Fisch. ex DC. [Fabaceae; Glycyrrhizae radix et rhizoma et rhizoma praeparata cum melle]. Jie et al. (2021) administered KJL throught gavage to treat DSS-induced rats, using mesalazine as a positive control. The results showed that both KJL and mesalazine effectively ameliorated colon injury in UC rats, and decreased the levels of NLRP3, apoptosis-related speck-like protein (ASC), caspase-1, gasdermin-D n-terminal domain (GSDMD-N), IL-1β, and IL-18. In addition, KJL also downregulated the expression of miR-223. Interestingly, in vitro experiments found that overexpression or inhibition of miR-223 did not consistently affect pyroptosis-related proteins, but consistently altered IL-1β mRNA levels, suggesting that KJL does not reduce UC by regulating miR-223 to inhibit pyroptosis. However, the mechanism through which KJL regulates miR-223 to reduce inflammation requires further investigation.

Qixianyijiang Decoction (QXYJD) is composed of 16 botanical drugs, such as *Astragalus mongholicus Bunge* [Fabaceae; Astragali radix], *Agrimonia eupatoria* L. [Rosaceae; agrimoniae herba], *Coix lacryma-jobi var. ma yuen* (Rom.Caill.) Stapf [Poaceae; Coicis semen], *Patrinia scabiosifolia* Link [Caprifoliaceae; dahurian patrinia]. Chen et al. (2022) used mesalazine in combinationed with a TCM enema to treat patients with spleen deficiency and dampness blockage-type UC inas the control group, while, and addedadministering QXYJD toas the observation group. The results revealedshowed that QXYJD could significantly improve patients’ clinical symptoms, such as diarrhoea, abdominal pain, and sticky bloody mucoid stool and so on, intestinal mucosal lesion, DAI, and the endoscopic range of motion (Baron) scores. QXYJD reduced the levels of the serum inflammatory indicators, such as C-reactive protein, IL-12, and IL-13, and downregulated the expression of miR-21-5p and miR-98-5p, suggesting that QXYJD potentially play an anti-inflammatory role by decreasing the expression of serum miR-21-5p and miR-98-5p; however, its specific mechanism is not clear.

Anchang Decoction (ACD) is composed of 16 botanical drugs, such as *C. corylifolium* (L.) Medik. [Fabaceae; psoraleae fructus], *A. mongholicus Bunge* [Fabaceae; Astragali radix], *Codonopsis pilosula* (Franch.) Nannf. [Campanulaceae; codonopsis radix]. Liang et al. (2021) intragastrically administered ACD to administrate 2, 4, 6-trinitrobenzenesulfonic acid (TNBS)/ethanol-induced rats by intragastric administration. Using as a positive control drug, both ACD and lLizonole effectively reduced the levels of TNF-α, IL-17, and other pro-inflammatory factors in the serum of rats and increased the levels of anti-inflammatory factor IL-10. The effect of ACD on the upregulation of IL-10 was greater than that of rezonolol. In animal experiments, ACD significantly decreased the relative expression of miR-146a in plasma and decreased the expression of IL-1 receptor-associated kinase 1 (IRAK1) and NF-κB protein in colon tissue, suggesting that ACD potentially plays an anti-inflammatory role in inhibiting the activation of the miRNA-146a/IRAK1/NF-κB pathway. However, this study did not further prove the relationship between miR-146a and IRAK1 expression.

#### 4.3.2 Metabolites of botanical drugs

Artesunate (ART) is a derivative extracted from *Artemisia annua* L. [Asteraceae; artemisiae annuae herba] and has antimalarial, anti-inflammatory, antioxidant, and antitumour properties (Zuo et al., 2016). Yang et al. (2021) found that ART could significantly inhibit the levels of inflammatory factors IL-12, IL-17, IL-23, and TNF-a in a dose-dependent manner in both *in vivo* and *in vitro* experiments, regardless of the mRNA level or serum level. Concurrently, ART downregulates the expression of miR-155 and the protein levels of p-NF-κB. Subsequent transfection experiments demonstrated that overexpression of miR-155 significantly increases the protein levels of p-NF-κB and reverses the effect of ART on NF-κB signalling, suggesting that ART inhibits UC progression by inhibiting inflammatory infiltration through inhibition of the miR-155/NF-κB axis.

Limonin is a triterpenoid extracted from *Citrus × aurantium* f. aurantium [Rutaceae; aurantii fructus]. Studies have revealed that limonin plays a significant role in promoting cancer cell death, regulating low-density lipoproteins, treating colon cancer, and fighting bacterial infections (Hamdan et al., 2011). Liu et al. (2019) administered limonin through gavage to interfere with DSS-induced mice, with Salazosulfapyridine used as a positive control. The results showed that both Limonin and Salazosulfapyridine significantly reduced the levels of pro-inflammatory factors IL-6 and TNF-α, especially in the medium-dose group. In subsequent experiments, the inhibitory effect of Limonin on STAT3 and miR-214 was confirmed. Overexpression of miR-214 weakened the anti-inflammatory ability of limonin. This study suggests that limonin reduces the expression of pro-inflammatory factors by inhibiting the signalling of STAT3/miR-214.

Ginsenoside (Rh2) is a proto-ginsenoside derived from *P. ginseng* C.A.Mey. [Araliaceae; Ginseng radix et rhizoma] that has anti-inflammatory and anticancer effects. Chen X. et al. (2021) administered Rh2 through gavage to interfere with DSS-induced mice, using salazosulapyridine as a positive control drug. The results showed that both Rh2 and Salazosulfapyridine effectively inhibited weight loss and colon shortening in UC mice. Rh2 decreased the levels of serum inflammatory factors IL-1β, IL-6, and TNF-α and downregulated the expression of STAT3 and miR-214. *In vitro* experiments revealed that Rh2 interfered with IL-6-induced NCM460 cells and significantly impaired the ability of miR-214 mimics to induce miR-214 overexpression and the concomitant downregulation of the downstream gene PTEN. This suggests that Rh2 may play a role in the treatment of UC by decreasing the levels of pro-inflammatory cytokines and related proteins in the STAT3/miR-214 signalling pathway.

BBR is an alkaloid extracted from *C. chinensis* Franch. [Ranunculaceae; coptidis rhizoma], Chen et al. (2024) demonstrated that the overexpression of miR-34a-5p could inhibit the viability of HT-29 cells, reduce the levels of IL-6, IL-1β, and TNF-α and downregulate the expression of IL-6 and STAT3. However, BBR intervention reversed these changes, indicating that BBR upregulated the expression of miR-34a-5p. Inhibiting the IL-6/STAT3 signalling pathway improves inflammation and alleviates UC.

It is apparent that Chinese medicine formulas and metabolites of botanical drugs play an irreplaceable role in repairing the UC-damaged intestinal mucosal barrier. By regulating specific miRNAs and targeting related signalling pathways and downstream proteins, the permeability of the intestinal mucosal barrier can be decreased, the tight connection of the intestinal mucosal barrier can be enhanced, and the expression of inflammatory factors in the UC colon tissue can be reduced. This helps maintain homeostasis of the immune system in UC, thereby reducing the recurrence rate and delaying the progression of UC lesions. In addition, TCM offers numerous advantages such as significantly improving clinical symptoms, enhancing patients’ quality of life, reducing adverse reactions, and minimising economic burdens.

## 5 Conclusion

Owing to the complex aetiology and recurrent symptoms of UC, developing innovative treatment strategies that can repair mucosal barrier function, promote deep mucosal healing, and effectively maintain remission is a major challenge. Recently, the role of miRNAs as diagnostic tools or therapeutic targets for UC has been widely studied. In this study, we used miRNAs as a focal point to investigate the regulation of miRNAs through Chinese medicine in the treatment of UC. Our findings indicate that Chinese medicine formulas and metabolites of botanical drugs can improve intestinal barrier damage in UC by regulating miRNAs. However, practical clinical application still faces obstacles. In particular, the mirNA-mediated post-transcriptional molecular networks that regulate the expression of many genes remain largely unknown. Rigorous therapeutic trials to study complex miRNA networks and their target genes are necessary to develop new interventions aimed at promoting deep mucosal healing. Therefore, future studies should explore the therapeutic effects and molecular mechanisms of TCM on UC through the regulation of miRNAs *in vivo* and *in vitro*, from multiple perspectives and methodologies. This approach will provide novel ideas and methods to clinically treat UC, highlighting the great advantages of TCM in the treatment of UC.

## Figures and Tables

**Table:** 

**UC**	Ulcerative colitis
**miRNAs**	MicroRNAs
**TCM**	Traditional Chinese medicine
**nts**	Nucleotides
**IGR**	Intron or gene spacer region
**pri-miRNAs**	Primary miRNAs
**pre-miRNA**	Precursor miRNA
**RISCs**	RNA-induced silencing complexes
**sIgA**	Secretory immunoglobulin A
**GALT**	Associated lymphoid tissue
**3′ UTR**	3′ untranslated region
**ISC**	Intestinal stem cells
**IECs**	Intestinal epithelial cells
**TJs**	Tight junctions
**ZO-1**	Tight junction protein 1
**JAM**	Junction adhesion molecule
**MUC2**	Recombinant Mucin 2
**IBD**	Inflammatory bowel disease
**HIF-1**	Hypoxia-inducible factor 1
**NLR**	NOD-like receptors
**PRR**	Pattern recognition receptors
**DCs**	Dendritic cells
**TLR4**	Toll-like receptor 4
**SMAR1**	Scaffold/matrix-associated region binding protein 1
**NF-κB**	Nuclear factor κB
**TNF-α**	Tumor Necrosis Factor-α
**IL-1β**	Interleukin-1β
**IL-6**	Interleukin-6
**IL-17**	Interleukin-17
**HT-29**	Human colon cancer cells
**STAT3**	Signal transducer and activator of transcription 3
**CAC**	Colitis-associated cancer
**CA**	Cinnamaldehyde
**BBR**	Berberine
**SMAR1**	Scaffold/stroma-associated region-binding protein 1
**VD**	Vitamin D
**MDA**	Malondialdehyde
**BRD4**	Bromodopamine-containing protein 4
**SOD**	Superoxide dismutase
**DAI**	Disease Activity Index
**BMDCs**	Bone marrow-derived dendritic cells
**NOTCH1**	Neurogenic locus notch homolog protein 1
**PPARγ**	Peroxisome proliferator-activated receptor

## Data Availability

The raw data supporting the conclusions of this article will be made available by the authors, without undue reservation.
