# Supplementary material for: MiRNAs: a new target for Chinese medicine to repair the intestinal barrier in the treatment of ulcerative colitis
Source: Front Pharmacol. 2024 Aug 9;15:1446554. doi: 10.3389/fphar.2024.1446554 (PMC11341499; doi:10.3389/fphar.2024.1446554)
Supplement: Supplementary file 1 [file Table1.DOCX]

TABLE 1. Lists of formula and metabolites of Chinese botanical drugs that reduce colonic permeability and repaire intestinal mucosal barrier integrity

| **Chinese medicine formula** | **Composition** | **miRNA** | **Model** | **Method of administration** | **Optimal dose** | **Positive control** | **Effects and potential mechanism** | **References** |
| --- | --- | --- | --- | --- | --- | --- | --- | --- |
| Shenlingbaizhu San | *Panax ginseng* C.A.Mey. [Araliaceae; Ginseng radix et rhizoma], *Poria cocos* (Schw.) Wolf [Polyporaceae; Poria], *Atractylodes macrocephala Koidz.* [Asteraceae; Atractylodis macrocephalae rhizoma], *Lablab purpureus subsp*. purpureus [Fabaceae, lablab semen album], *Dioscorea oppositifolia L.* [Dioscoreaceae, dioscoreae rhizoma], *Glycyrrhiza uralensis* Fisch. ex DC. [Fabaceae; Glycyrrhizae radix et rhizoma], *Nelumbo nucifera Gaertn.* [Nelumbonaceae; Nelumbinis semen], *Wurfbainia villosa* (Lour.) Škorničk. and A.D.Poulsen [Zingiberaceae; Amomi fructus], *Coix lacryma-jobi var. ma- yuen* (Rom.Caill.) Stapf [Poaceae; Coicis semen], *Platycodon grandiflorus* (Jacq.) A.DC. [Campanulaceae; Platycodonis radix], *Ziziphus jujuba* Mill. [Rhamnaceae; jujubae fructus] | miR-130a↓ | DSS induced  mice | oral gavage | 12.13g/kg | Mesalazine | down-regulating miR-130a expression, enhancing the expression of colon tight junctions (TJs) protein and improving intestinal epithelial barrier damage | Zhu, 2022b |
| Kaixuan Decoction | *Codonopsis pilosula* (Franch.) Nannf. [Campanulaceae; codonopsis radix], *Hansenia weberbaueriana* (Fedde ex H.Wolff) Pimenov & Kljuykov [Apiaceae; notopterygii rhizoma et radix], *Angelica biserrata* (R.H.Shan & C.Q.Yuan) C.Q.Yuan & R.H.Shan [Apiaceae; angelicae pubescentis radix], *Astragalus mongholicus Bunge* [Fabaceae; Astragali radix], *Bupleurum chinense* DC. [Apiaceae; bupleuri radix], *Kitagawia praeruptora* (Dunn) Pimenov [Apiaceae], *Atractylodes macrocephala Koidz. [Asteraceae; Atractylodis macrocephalae rhizoma]*, *Citrus × aurantium* f. aurantium [Rutaceae; Aurantii fructus], *Platycodon grandiflorus* (Jacq.) A.DC. [Campanulaceae; platycodonis radix], *Actaea cimicifuga* L. [Ranunculaceae; cimicifugae rhizoma], *Saposhnikovia divaricata* (Turcz. ex Ledeb.) Schischk. [Apiaceae; saposhnikoviae radix], *Conioselinum anthriscoides 'Chuanxiong'* [Apiaceae; chuanxiong rhizoma], Gentiana macrophylla Pall. [Gentianaceae; gentianae macrophyllae radix], *Poria cocos* (Schw.) Wolf [Polyporaceae; Poria], *Glycyrrhiza uralensis* Fisch. ex DC. [Fabaceae; Glycyrrhizae radix et rhizoma], *Wurfbainia villosa* (Lour.) Škorničk. and A.D.Poulsen [Zingiberaceae; Amomi fructus], *Dendrobium nobile* Lindl. [Orchidaceae; dendrobii caulis], *Mentha canadensis* L. [Lamiaceae; menthae haplocalycis herba] | miR-155↓  miR-195↑ | Patients with mild UC | oral | - | Live Combined Bacillus Subtilis and Enterococcus Faecium Enteric-coated Capsules combined withSulfasalazine Enteric-coated Tablets | down-regulating miR-155 expression and up-regulating miR-195 expression, reducing levels of intestinal mucosal barrier function indicators (D-LA, BT, DAO), and repairing the intestinal mucosal barrier | Qiu et al., 2022 |
| Fufangkushen Decoction | *Sophora flavescens* Aiton [Fabaceae; sophorae flavescentis radix],  *Sanguisorba officinalis* L. [Rosaceae; carbonized sanguisorbae radix], *Bletilla striata* (Thunb.) Rchb.f. [Orchidaceae; bletillae rhizoma], *Glycyrrhiza uralensis* Fisch. ex DC. [Fabaceae; Glycyrrhizae radix et rhizoma], *Panax notoginseng* (Burkill) F.H.Chen [Araliaceae; notoginseng radix et rhizoma], *Strobilanthes cusia* (Nees) Kuntze [Acanthaceae; indigo naturalis] | miR-146a↑ | DSS induced  mice | oral gavage | 14.56 g/kg | Mesalazine | increasing miR-146a expression, inhibiting the excessive activation of the Notch signaling pathway, exerting anti-inflammatory, anti-apoptosis and proliferation, enhancing TJs, reducing intestinal permeability, promoting goblet cell differentiation and mucus secretion functions, thus repairing the intestinal mucosal barrier | Wu, 2022 |
| Qingchangwenzhong Formula | *Coptis chinensis* Franch. [Ranunculaceae; coptidis rhizoma], *Zingiber officinale* Roscoe [Zingiberaceae; zingiberis rhizoma praeparatum], *Strobilanthes cusia* (Nees) Kuntze [Acanthaceae; indigo naturalis], *Sophora flavescens* Aiton [Fabaceae; sophorae flavescentis radix], *Panax notoginseng* (Burkill) F.H.Chen [Araliaceae; notoginseng radix et rhizoma], *Dolomiaea costus* (Falc.) Kasana & A.K.Pandey [Asteraceae; aucklandiae radix], *Sanguisorba officinalis* L. [Rosaceae; carbonized sanguisorbae radix], *Glycyrrhiza uralensis* Fisch. ex DC. [Fabaceae; Glycyrrhizae radix et rhizoma] | miR-675-5p↓ | DSS induced  mice | oral gavage | - | - | down-regulating miR-675-5p expression, targeting VDR signaling pathway, enhancing the expression of colon TJs protein, and improving intestinal epithelial barrier damage | Sun et al., 2019 |
| Baicalin | *Scutellaria baicalensis* Georgi [Lamiaceae; scutellariae radix] | miR-191a↓ | TNF-α induced IEC-6 cells | added to the culture medium | 40 μg/mL | - | down-regulating miR-191a, targeting ZO-1, reducing colon permeability, and restoring the integrity of the intestinal epithelial barrier. | Wang, 2018 |
| Icariin | *Epimedium sagittatum* (Siebold & Zucc.) Maxim. [Berberidaceae; epimedii folium] | miR-122↓ | TNF-α induced Caco-2 cells | added to the culture medium | 5μM |  | down-regulating miR-122, targeting Occludin, reducing colon permeability, repairing the intestinal mucosal barrier | Yu et al., 2023 |
| Matrine | *Sophora flavescens* Aiton [Fabaceae; sophorae flavescentis radix] | miR-155↓ | Caco-2 cells overexpressing miR-155 | added to the culture medium | - | - | down-regulating miR-155, inhibiting related protein of Rho/Rock pathwat- ROCK1, and keeping TJ, thus repairing the intestinal mucosal barrier | Yu et al., 2023 |
|  |  |  | DSS induced mice | oral gavage | 20 mg/kg | - |  |  |
| Berberine | *Coptis chinensis* Franch. [Ranunculaceae; coptidis rhizoma], *Phellodendron chinense* C.K.Schneid. [Rutaceae; phellodendri chinensis cortex], Berberis vulgaris L. [Berberidaceae; berberidis radix] | miR-103a-3p↑ | DSS induced mice | oral gavage | 100 mg/kg | - | up-regulating miR-103a-3pdown-regulating , targeting BRD4, inhibiting Wnt/β-catenin pathway, up-regulating Occludin, repairing the intestinal mucosal barrier | Zhao, 2023 |
| Naringenin | *Citri Reticulatae* Blanco [Rutaceae; citri reticulatae pericarpium],  *Citrus × aurantium f. aurantium* [Rutaceae; aurantii fructus immaturus] | miR-22↑ | DSS induced mice | intraperitoneal injection | 100 mg/kg | - | up-regulating miR-22, thus inactivating NLRP3 inflammation complex, enhancing the ability of TJs proteins, and repairing the intestinal epithelial barrier | Xie et al., 2021 |

TABLE 2. Lists of formula and metabolites of Chinese botanical drugs that regulate intestinal immune balance and alleviate damage to the intestinal mucosal barrier

| **Chinese medicine formula** | **Composition** | **miRNA** | **Model** | **Method of administration** | **Optimal dose** | **Positive control** | **Effects and potential mechanism** | **References** |
| --- | --- | --- | --- | --- | --- | --- | --- | --- |
| Sishen Pill | *Cullen corylifolium* (L.) Medik. [Fabaceae; psoraleae fructus], *Tetradium ruticarpum* (A.Juss.) T.G.Hartley [Rutaceae; euodiae fructus], *Myristica fragrans* Houtt. [Myristicaceae; myristicae semen], *Schisandra chinensis* (Turcz.) Baill. [Schisandraceae; schisandrae chinensis fructus], *Zingiber officinale* Roscoe [Zingiberaceae; zingiberis rhizoma recens], *Ziziphus jujuba* Mill. [Rhamnaceae; jujubae fructus] | miR-505-3p↓ | DSS induced  mice | oral gavage | 5g/kg | - | down-regulating miR-505-3p, inhibiting the expression of E-cadherin protein, avoiding the cascade amplification of inflammation, and regulate intestinal immune homeostasis | Huang, 2024 |
|  |  |  | LPS induced bone marrow-derived dendritic cells of mice | added to the culture medium | 4 mg/mL | - | limiting the miR-505-3p to suppress the dendritic cells on E - cadherin protein expression, thus inhibiting dendritic cells to the inflammatory state of differentiation |  |
| Xiezhuojiedu Formula | *Houttuynia cordata* Thunb. [Saururaceae; houttuyniae herba], *Plantago ovata* Forssk. [Plantaginaceae; plantaginis semen], *Scutellaria baicalensis* Georgi [Lamiaceae; scutellariae radix], *Coptis chinensis* Franch. [Ranunculaceae; coptidis rhizoma], *Patrinia scabiosifolia* Link [Caprifoliaceae; herba patriniae], *Sargentodoxa cuneata* (Oliv.) Rehder & E.H.Wilson [Lardizabalaceae; sargentodoxae caulis], *Pueraria montana* var. lobata (Willd.) Maesen & S.M.Almeida ex Sanjappa & Predeep [Fabaceae; puerariae lobatae radix], *Coix lacryma-jobi var. ma- yuen* (Rom.Caill.) Stapf [Poaceae; Coicis semen], *Dolomiaea costus* (Falc.) Kasana & A.K.Pandey [Asteraceae; aucklandiae radix], *Pinellia ternata* (Thunb.) Makino [Araceae] | miR-155-5p↓ | DSS induced  mice | oral gavage | 38.48g/kg | Mesalazine | down-regulating miR-155-5p expression, targeting the JAK2/STAT3/SOCS1 signaling pathway, inhibiting the expression of CD4 T cells to Th17 cells | Sun et al., 2024 |
| Qingchang Huayu Formula | Pulsatilla chinensis (Bunge) Regel [Ranunculaceae; pulsatillae radix], *Coptis chinensis* Franch. [Ranunculaceae; coptidis rhizoma], *Fraxinus excelsior* L. [Oleaceae, fraxini cortex], *Taraxacum sect. Taraxacum* F.H.Wigg. [Asteraceae; taraxaci herba], Forsythia suspensa (Thunb.) Vahl [Oleaceae; forsythiae fructus], *Sophora flavescens* Aiton [Fabaceae; sophorae flavescentis radix], *Bletilla striata* (Thunb.) Rchb.f. [Orchidaceae; bletillae rhizoma], *Portulaca oleracea* L. [Portulacaceae; portulacae herba], *Patrinia scabiosifolia* Link [Caprifoliaceae; herba patriniae], *Styphnolobium japonicum* (L.) Schott [Fabaceae; sophorae flos], *Trichosanthes kirilowii* Maxim. [Cucurbitaceae; trichosanthis radix], *Sanguisorba officinalis* L. [Rosaceae; sanguisorbae radix], *Salvia miltiorrhiza* Bunge [Lamiaceae; Salviae miltiorrhizae radix et rhizoma], *Angelica sinensis* (Oliv.) Diels [Apiaceae; angelicae sinensis radix], *Astragalus mongholicus Bunge* [Fabaceae; Astragali radix],*Dolomiaea costus* (Falc.) Kasana & A.K.Pandey [Asteraceae; aucklandiae radix], *Panax notoginseng* (Burkill) F.H.Chen [Araliaceae; notoginseng radix et rhizoma] | miR-22↓ | patients with damp heat of the large intestine syndrome in UC | oral | - | Mesalazine | down-regulating miR-22 and inhibiting the inflammatory differentiation of Th17 cells | Yang et al., 2022 |
| Jiuweibaizhu Decoction | *Pueraria montana* var. lobata (Willd.) Maesen & S.M.Almeida ex Sanjappa & Predeep [Fabaceae; puerariae lobatae radix], *Sargentodoxa cuneata* (Oliv.) Rehder & E.H.Wilson [Lardizabalaceae; sargentodoxae caulis], *Codonopsis pilosula* (Franch.) Nannf. [Campanulaceae; codonopsis radix], *Atractylodes macrocephala Koidz.* [Asteraceae; Atractylodis macrocephalae rhizoma], *Poria cocos* (Schw.) Wolf [Polyporaceae; Poria], Pogostemon cablin (Blanco) Benth. [Lamiaceae; pogostemonis herba], *Bletilla striata* (Thunb.) Rchb.f. [Orchidaceae; bletillae rhizoma], *Dolomiaea costus* (Falc.) Kasana & A.K.Pandey [Asteraceae; aucklandiae radix], *Glycyrrhiza uralensis* Fisch. ex DC. [Fabaceae; Glycyrrhizae radix et rhizoma] | miR-155↓ | TNBSl induced rats | oral gavage and enteric cavity injection | 32. 55 g / kg | overexpress miR-155 | Inhibiting miR- 155 expression and promoting SOCS - 1 protein expression, thus inhibiting proinflammatory factor signal transduction and intestinal mucosal immune function factor expression | Yang et al., 2023 |
| Fufangkushen Decoction | Refer to TABLE 1. | miR-155↓ | DSS induced  mice | oral gavage and enteric cavity injection | 0.2ml | Mesalazine | down-regulating miR-155, targeting Jarid2/Wnt/β-catenin axis to regulate the expression of Th17 and Treg cells and their related inflammatory factors | Zhu, 2022a |
|  |  |  | naïve CD4 +T cells of spleen in mice | added to the culture medium | 2ml | miR-155 inhibitor |  |  |
| Curcumin | *Curcuma longa* L. [Zingiberaceae; curcumae longae rhizoma] | miR-425↓ | TNBS induced rats | oral gavage | 100mg/kg | - | down-regulating miR-425, regulating Th17/Treg balance, maintaining intestinal immune homeostasis | Song et al., 2020 |
| Resveratrol | *Reynoutria japonica* Houtt. [Polygonaceae; polygoni cuspidati rhizoma et radix], *Smilax glabra* Roxb. [Smilacaceae; Smilacis glabrae rhizoma] | miR-31↓ | TNBS induced mice | oral gavage | 100mg/kg | - | down-regulating miR-31, targeting Foxp3 pathway, regulating Th17/Treg balance | Alrafas et al., 2020 |
| Alpinetin | *Curcuma longa* L. [Zingiberaceae; curcumae longae rhizoma], *Wurfbainia compacta* (Sol. ex Maton) Škorničk. & A.D.Poulsen [Zingiberaceae; amomi fructus rotundus], *Curcuma longa* L. [Zingiberaceae; curcumae radix] | miR-302↑ | DSS induced mice | oral gavage | 30 mg/kg | Mesalazine | regulating miR-302/DNMT-1/CREB pathway, improving immune disorders, reducing inflammatory infiltration | Lv et al., 2018) |
|  |  |  | naïve CD4 +T cells of mesenteric lymph nodes in mice | added to the culture medium | 30 μM | - |  |  |
| Cinnamaldehyde | *Cinnamomum verum* J.Presl [Lauraceae; cinnamomi cortex] | miR-21↓  miR-155↓ | DSS induced mice | oral gavage | 10mg/kg | Mesalazine | inhibiting miR-21 of macrophage  cells and down-regulating miR-155, regulating AKT/mTOR and COX2 pathway，thus regulating immune, reducing inflammatory infiltration | Qu et al., 2019 |
|  |  |  | LPS induced RAW.264.7 cells | added to the culture medium | 20 μM | - |  |  |
| Diosgenin | *Dioscorea nipponica* Makino [Dioscoreaceae; dioscoreae nipponicae rhizoma], *Dioscorea nipponica* Makino [Dioscoreaceae; dioscoreae rhizoma] | miR-125a-5p↑ | DSS induced mice | oral gavage | 80 mg/kg | - | up-regulating miR-125a-5p and regulating macrophage polarization | Shi et al., 2022 |

TABLE 3. Lists of formula and metabolites of Chinese botanical drugs that reduce inflammation and protect colonic mucosa

| **Chinese medicine formula** | **Composition** | **miRNA** | **Model** | **Method of administration** | **Optimal dose** | **Positive control** | **Effects and potential mechanism** | **References** |
| --- | --- | --- | --- | --- | --- | --- | --- | --- |
| Gegen Qinlian Decoction | *Pueraria montana var. lobata* (Willd.) Maesen & S.M.Almeida ex Sanjappa & Predeep [Fabaceae; puerariae lobatae radix], *Coptis chinensis* Franch. [Ranunculaceae; coptidis rhizoma], *Scutellaria baicalensis* Georgi [Lamiaceae; scutellariae radix], *Glycyrrhiza uralensis* Fisch. ex DC. [Fabaceae; Glycyrrhizae radix et rhizoma] | miR-542-3p↑ | DNCB/ ethyl alcohol/acetic acid induced rats | oral gavage | 12g/kg | Mesalazine | up-regulating miR-542-3p, inhibiting inflammatory mediators and reducing oxidative stress | Gong et al., 2022) |
| Kuijieling Decoction | Ilex rotunda Thunb. [Aquifoliaceae, ilicis rotundae cortex], *Atractylodes lancea* (Thunb.) DC. [Asteraceae, rhizoma atractylodis macrocephalae], *Paeonia lactiflora Pall.* [Paeoniaceae; Paeoniae radix rubra], Hirudo nipponica Whitman [Hirudinidae; Hirudo], *Glycyrrhiza uralensis* Fisch. ex DC. [Fabaceae; Glycyrrhizae radix et rhizoma et rhizoma praeparata cum melle] | miR-223↓ | DSS induced mice | oral gavage | 9.2 g/kg | Mesalazine | down-regulating miR-223, inhibiting intestinal inflammation | Jie et al., 2021 |
|  |  |  | RAW264.7 cells | added to the culture medium | 10%含KJL清 | - |  |  |
| Qixianyijiang Decoction | *Astragalus mongholicus Bunge* [Fabaceae; Astragali radix], *Agrimonia eupatoria* L. [Rosaceae; agrimoniae herba], *Coix lacryma-jobi var. ma- yuen* (Rom.Caill.) Stapf [Poaceae; Coicis semen], *Patrinia scabiosifolia* Link [Caprifoliaceae; dahurian patrinia], *Platycodon grandiflorus* (Jacq.) A.DC. [Campanulaceae; platycodonis radix], *Prunus mume* (Siebold) Siebold & Zucc. [Rosaceae; mume fructus], *Angelica sinensis* (Oliv.) Diels [Apiaceae; angelicae sinensis radix], *Dolomiaea costus* (Falc.) Kasana & A.K.Pandey [Asteraceae; aucklandiae radix],*Coptis chinensis* Franch. [Ranunculaceae; coptidis rhizoma], *Sargentodoxa cuneata* (Oliv.) Rehder & E.H.Wilson [Lardizabalaceae; sargentodoxae caulis], *Toona sinensis* (A.Juss.) M.Roem. [Meliaceae, ailanthi cortex], *Sophora flavescens* Aiton [Fabaceae; sophorae flavescentis radix], *Sanguisorba officinalis* L. [Rosaceae; carbonized sanguisorbae radix],  *Nepeta tenuifolia* Benth. [Lamiaceae; schizonepetae herba carbonisata],  *Atractylodes lancea* (Thunb.) DC. [Asteraceae; fried atractylodis rhizoma], *Atractylodes lancea* (Thunb.) DC. [Asteraceae, fried rhizoma atractylodis macrocephalae] | miR-21-5p↓  miR-98-5p↓ | patients with spleen deficiency and dampness obstruction syndrome | oral | - | Mesalazine | down-regulating miR-21-5p and miR-98-5, playing an anti-inflammatory role | Chen et al., 2022 |
| Anchang Decoction | *Cullen corylifolium* (L.) Medik. [Fabaceae; psoraleae fructus], *Astragalus mongholicus Bunge* [Fabaceae; Astragali radix], *Codonopsis pilosula* (Franch.) Nannf. [Campanulaceae; codonopsis radix], *Zingiber officinale* Roscoe [Zingiberaceae; zingiberis rhizoma], *Atractylodes lancea* (Thunb.) DC. [Asteraceae, fried rhizoma atractylodis macrocephalae], *Pulsatilla chinensis* (Bunge) Regel [Ranunculaceae; pulsatillae radix], *Poria cocos* (Schw.) Wolf [Polyporaceae; Poria], Areca catechu L. [Arecaceae; arecae semen], *Aconitum carmichaelii Debeaux* [Ranunculaceae; Aconiti lateralis radix praeparata], Gallus gallus domesticus Brisson [Galli gigerii endothelium corneum], *Coix lacryma-jobi var. ma- yuen* (Rom.Caill.) Stapf [Poaceae; Coicis semen], *Dolomiaea costus* (Falc.) Kasana & A.K.Pandey [Asteraceae; aucklandiae radix], *Sanguisorba officinalis* L. [Rosaceae; sanguisorbae radix], Paeonia lactiflora Pall. [Paeoniaceae, paeoniae radix rubra], Corydalis yanhusuo (Y.H.Chou & Chun C.Hsu) W.T.Wang ex Z.Y.Su & C.Y.Wu [Papaveraceae; corydalis rhizoma], *Glycyrrhiza uralensis* Fisch. ex DC. [Fabaceae; Glycyrrhizae radix et rhizoma et rhizoma praeparata cum melle] | miR-146a↓ | TNBS/ ethyl alcohol induced rats | oral gavage | 5ml / kg | Bifidobacterium | down-regulating miRNA-146a, inhibiting activation of IRAK1/NF-κB pathway, playing an anti-inflammatory role | Liang et al., 2021 |
| Artesunate | *Artemisia annua* L. [Asteraceae; artemisiae annuae herba] | miR-155↓ | LPS induced RAW264.7 cells | added to the culture medium | 160 μg/mL | - | down-regulating miR-155, inhibiting NF-κB pathway activation, thus inhibiting inflammatory infiltration | Yang et al., 2021 |
|  |  |  | TNBS/ ethyl alcohol induced mice | oral gavage | 150 mg/kg |  |  |  |
| Limonin | *Citrus × aurantium* f. aurantium [Rutaceae;  aurantii fructus] | miR-214↓ | DSS induced mice | oral gavage | 80 mg/kg | Salazosulfapyridine | inhibiting STAT3/miR-214 signal transduction, thus reducing pro-inflammatory factors | Liu et al., 2019 |
|  |  |  | IL-6 induced NCM460 cells | added to the culture medium | 40 μg/mL | - |  |  |
| Ginsenoside Rh2 | *Panax ginseng* C.A.Mey. [Araliaceae; Ginseng radix et rhizoma] | miR-214↓ | DSS induced mice | oral gavage | 50 mg/kg | Salazosulfapyridine | reducing levels of pro-inflammatory cells factors and related proteins in STAT3/miR-214 signal pathway | Chen et al., 2021b |
|  |  |  | IL-6 induced NCM460 cells | added to the culture medium | 10 μM/L | - |  |  |
| Berberine | Refer to TABLE 1. | miR-34a-5p↑ | LPS induced HT-29 cells | added to the culture medium | 20 μg/mL | - | up-regulating miR-34a-5p, inhibiting IL-6/STAT3 signal pathway, thus improving inflammation levels | Chen et al., 2024) |
